# Reinforcement learning for proposing smoking cessation activities that build competencies: Combining two worldviews in a virtual coach

**DOI:** 10.1186/s12911-025-03164-8

**Published:** 2025-10-10

**Authors:** Nele Albers, Mark A. Neerincx, Willem-Paul Brinkman

**Affiliations:** 1 Department of Intelligent Systems, Delft University of Technology, Delft, Netherlands; 2 Department of Communication and Cognition, Tilburg University, Tilburg, Netherlands

**Keywords:** Behavior change, Conversational agent, eHealth, Mental model, Persuasion, Psychology-informed algorithm, Reinforcement learning, Just-in-time adaptive intervention

## Abstract

**Background:**

Reaching personal goals typically requires building competencies (e.g., insights into personal strengths), but expert health professionals and non-expert clients often think differently about which competencies are needed. Just having a virtual coach advise activities for "expert-devised" competencies may not motivate clients to carry them out, while advising only "non-expert devised" activities may not result in all required competencies being built.

**Methods:**

We integrated the client and health expert worldviews in our modeling method for informing the activity selection by a virtual coach: We created a pipeline to build a reinforcement learning model for proposing activities in the context of preparing for quitting smoking. This model considers smokers’ current and future levels for expert-devised competencies as well as their beliefs about the usefulness of different competencies when choosing activities. To train the model, we conducted a micro-randomized trial in which 542 smokers interacted with a virtual coach in five sessions spread over at least nine days and received a randomly chosen activity in each session. Using data from this study, we performed simulations to systematically assess the impact of the different model components on the competencies built by smokers. Moreover, we performed paired Bayesian *t*-tests to determine the effect of persuasive activities on smokers’ usefulness beliefs.

**Results:**

Our simulations show that smokers’ current levels for the expert competencies and their usefulness beliefs are important to consider when building expert competencies. In fact, we saw improvements of up to 22% when considering current competencies, and an additional 13% when also accounting for usefulness beliefs. Furthermore, although we found credible evidence that persuasive activities changed smokers’ usefulness beliefs, the effects might be too small to contribute in an optimal strategy for building competencies.

**Conclusion:**

The worldviews of both health experts and smokers are important to consider when proposing activities for preparing for quitting smoking. We have presented a reinforcement learning model that combines these worldviews, and we hope that our work can be an example of incorporating different worldviews in a reinforcement learning model for building competencies. Our code and dataset are publicly available.

## Introduction

Considering that 14.0% of the disease burden in the Netherlands stems from unhealthy behavior [1], coupled with the projection that by 2060, one in three Dutch workers will need to work in healthcare to cater to the aging population [2], eHealth applications have a large potential in supporting people in changing behaviors such as physical inactivity, unhealthy eating, and low-quality sleep [3]. Since smoking alone causes 7.6% of the Dutch disease burden [1], applications supporting smoking cessation [4, 5] are especially welcome. To increase engagement, discuss relevant information, and form a connection with people [6, 7], such eHealth applications commonly integrate conversational agents that take the role of virtual coaches guiding people through the behavior change intervention. For example, a virtual coach may propose activities such as envisioning one’s desired future self after quitting smoking, tracking one’s smoking behavior, or creating a motivational slogan. While meta-analyses have found initial evidence of overall positive effects and high acceptance of such guidance for both smoking cessation [6] and other contexts such as mental health [8], long-term engagement with and effectiveness of virtual coaches is still a challenge [6, 8]. Personalizing the guidance offered by the virtual coaches by providing the right support at the right time might be a way to address this. Such just-in-time adaptive interventions (JITAIs) have previously shown promise in various behavior change domains [9]. Here, we investigate how a virtual coach should decide which smoking cessation activities to propose. Our focus thereby is on activities for *preparing* for quitting smoking for two reasons. Not only is a preparation phase often included in smoking cessation interventions (e.g., [10–12]) to increase the chance of successful behavior change thereafter, but sub-optimal activity choices are also less risky to study when preparing for quitting smoking than when actually quitting. Following the stages in the development of technological health interventions defined by Brinkman [13], the most promising ways of proposing activities can then be tested in a full smoking cessation intervention in the future.

The virtual coach ultimately wants to propose activities that allow people to reach their behavioral goals. This often requires building competencies, such as being able to perform a breathing exercise, knowing what constitutes a healthy diet, or having self-confidence. These competencies are characteristics (e.g., knowledge, skills, mindsets, thought patterns) that when used, alone or together, result in successful behavior [14]. Following a means-end problem-solving approach, when people select subgoals (i.e., "means") to reach their goals [15, 16], the competencies that they perceive to be important are obvious "means" candidates. A person’s conscious (sub)goals affect the actions they take [17] and, consequently, knowing them helps to predict the subjective usefulness of the related action. As expressed by theories such as the Unified Theory of Acceptance and Use of Technology (UTAUT) [18], these perceptions of usefulness in turn affect a person’s effort investment (e.g., if a person thinks that practical knowledge will help them more to reach their goal of quitting smoking than physical fitness, they are likely to spend more effort on knowledge building than on physical activity). So knowing a person’s subgoals can help a virtual coach propose actions (i.e., activities) that the person is likely to spend effort on, and that will thus build the person’s competencies.

However, people do not always know which competencies are required for reaching a goal. According to the Dunning-Kruger effect [19], for example, people with little experience or knowledge regarding a task tend to overestimate their competence (e.g., because similar competencies to reach a goal are also needed to assess one’s performance [19]). Thus, when selecting subgoals, people may select different ones than experts would. This means that if the virtual coach would simply propose activities that people regard as useful, people might never build the competencies that experts consider relevant.

Our aim was thus to develop a model for informing a virtual coach’s selection of activities that build people’s competencies from the perspective of experts while accounting for the fact that if people do not find an activity useful, they are unlikely to do it thoroughly. People may need to first be convinced of its usefulness. Our model hence needs to consider which competencies people find useful (because people are more likely to do activities that build competencies they find useful) and the degree to which they have built the expert competencies (because we want to choose activities that ultimately help people build the expert-identified competencies). And since activities chosen at one point in time can influence future usefulness beliefs and degrees of having built expert competencies and thus which activities are effective in the future, our model needs to account for both current and future usefulness beliefs and degrees of having built the expert competencies if the activity selection should be effective in the long run. One framework that allows us to formulate a model accounting for such current and future states is Reinforcement Learning (RL). RL for adaptive behavior change support [20] with consideration of current and future states has previously been applied to send running notifications [21], suggest step goals [22, 23], recommend diabetes coaching interventions [24], or choose persuasive strategies for preparing for quitting smoking [25]. Here, we investigate how RL can be used to account for two worldviews when choosing activities that build human competencies for quitting smoking. Our overarching research question thus is

*How can we build an RL model for building human competencies for quitting smoking that combines the views of experts and smokers?*

**Fig. 1:**
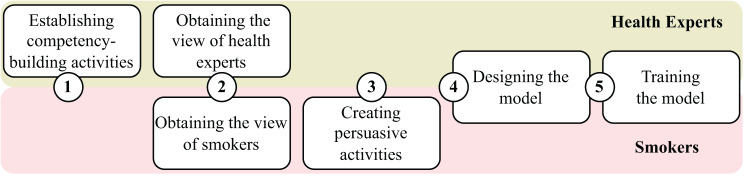
Pipeline for creating an RL model for proposing smoking cessation activities that build competencies for quitting smoking by accounting for the views of health experts and smokers

Our pipeline for creating such an RL model consisted of the five steps shown in Fig. 1: 1) Establishing competency-building activities as the actions that health experts recommend to reach the goal of quitting smoking, 2) obtaining the views of health experts and smokers that describe which competencies they think are built by these activities (i.e., expert-identified vs. smoker-identified competencies), 3) creating persuasive activities that can persuade smokers of the usefulness of smoker-identified competencies (e.g., by pointing out how the competency "motivation to change" can help to deal with withdrawal symptoms and nicotine cravings), 4) designing an RL model for proposing activities that optimizes the degree to which smokers build the expert-identified competencies while considering that the effort smokers spend on activities depends on which smoker-identified competencies they perceive as useful, and 5) training the model with data from a crowdsourcing study. Afterward, we evaluated the model by examining the effectiveness of its different components in building expert competencies and in changing smokers’ usefulness beliefs using human data-based simulations, which is a common way to evaluate RL models [20, 26].

This paper contributes insights into the effects of subjective usefulness beliefs and of possibilities to change them with short persuasive activities. These highlight the importance of accounting for people’s current worldviews rather than trying to change them when striving to build people’s competencies. Furthermore, we provide a model for proposing competency-building activities for quitting smoking which combines the views of health experts and smokers. This model alone is not a complete behavior change intervention. Instead, it can be used to personalize elements of both face-to-face and digital smoking cessation interventions, specifically the recommendation of activities. To facilitate this, we have made the dataset used to train our model and our activities publicly available in the online repository accompanying this paper [27]. Lastly, we hope that other researchers wishing to incorporate different worldviews in a reinforcement learning model for building competencies can use our pipeline as inspiration.

## Background

### Persuasive strategies in eHealth applications for behavior change

Providing behavior change support over the Internet or connected technologies such as apps and text messaging, eHealth applications for behavior change commonly ask their users to do activities such as designing motivational slogans, learning about nicotine replacement therapy, or reflecting on the past week. Persuasive strategies are often used to motivate people to do these activities. Several sets of persuasive strategies have been outlined. Oinas-Kukkonen and Harjumaa [28], for instance, identify persuasive strategies such as social learning and cooperation. Cialdini [29] introduces six persuasive strategies such as consensus, while Fogg [30] distinguishes between persuasive strategies associated with "technology as a tool" (e.g., self-monitoring) and those linked to "technology as a social actor" (e.g., language cues). Consolvo et al. [31] provide nine persuasive strategies, including aesthetics. It is worth noting that many of these persuasive strategies can be applied in various ways, such as framing messages differently (e.g., [32, 33]) and using different communication methods (e.g., [34, 35]).

### Algorithms for adaptive persuasive attempts

When applying these persuasive strategies, using a one-size-fits-all approach is unlikely to have a large effect on behavior [36, 37], as behavior change theories [38] suggest that many personal factors influence behavior. Using these factors as inspiration, previous work has developed algorithms for adapting *how* people are persuaded, *when*, and *by whom*. Work on the former includes adapting persuasive strategies to dynamic factors (e.g., people’s states derived from the COM-B model [25, 39], self-efficacy [40]) as well as more stable personal characteristics (e.g., personality, gender, and stage of change [41], age, gender, and personality [42]). Algorithmic techniques thereby range from RL (e.g., [23, 25, 43]) to recommender systems (e.g., [44]) and logistic regression (e.g., [45]). Dynamic factors have also been considered to optimize the timing and sender of persuasive attempts, for example in RL models for sending notifications for physical activity [21] and oral self-care [46] or deciding on the degree of human involvement in an intervention for chronic pain [47]. Yet, the effects of these approaches on behavior are typically small (e.g., [25, 48, 49]).

### Proposing useful activities

One reason for these small effects is that people do not necessarily find what is proposed useful. For example, Albers et al. [25] observed a large effect for personal relevance, involvement, and personal interest on the effort spent on activities for quitting smoking, in contrast to a small effect of adapting *how* people were persuaded to do the activities. Moreover, Faber et al. [50] recommend that especially eHealth applications for people with low socioeconomic status should be designed to be perceived as useful by the target group. This is in line with the algorithmic acceptance model [51], which posits that besides convenience, usefulness predicts people’s attitude toward an algorithm system and thus its actual use. Moreover, the related notion of performance expectancy is also one of the main predictors of the intention to use technology in the UTAUT [18]. Similarly, in the COM-B model [52] in which a person’s capability, opportunity, and motivation influence their behavior, analytical decision-making is one of the factors directing behavior.

Several previous works have thus optimized *what* is proposed to people. Costa et al. [53], for instance, select activities for elderly people by generating arguments in support of the activities and deciding which would be preferred by a person based on data from previous interactions. And Klein et al. [54] address a person’s bottlenecks for behavior change (e.g., attitude) based on urgency and the degree to which they can be changed. Yet, these approaches consider only what is useful objectively or from the perspective of *experts*, not what is useful from the perspective of *users*. Users’ usefulness beliefs, however, do not necessarily match those of experts. Although physical activity can make it easier to quit smoking [55, 56], for example, smokers do not necessarily consider physical activity useful to quit smoking [57]. Thus, while we ultimately want users to do activities that are perceived as useful by experts (i.e., build the competencies experts consider relevant), we need to account for users’ perceptions of usefulness. Given that RL allows us to consider people’s degrees of having built the expert competencies as well as their usefulness beliefs both currently and in the future, our first analysis question, therefore, is the following:

*AQ1: How effective is an RL model that combines the views of experts and smokers in building expert-identified competencies?*

### Changing beliefs

Rather than just *considering* people’s usefulness beliefs, one can also try to *change* them. This is especially important when the virtual coach can otherwise not build all expert competencies (e.g., because people find none of the related activities useful). Yet, changing beliefs can be difficult because people attribute importance to their beliefs and are hence prepared to act on and hold to these beliefs even when presented with conflicting evidence, especially when the beliefs are strong [58]. From the perspective of conceptual change, learners bring conceptions constructed from their own experiences with them that are potentially incorrect from the standpoint of established knowledge and thus hinder learning [58]. When such misconceptions exist, learning requires changes in learners’ personal mental models or representations. This is because information that does not fit the learners’ mental models is ignored or misunderstood [59]. Any of the persuasive strategies defined earlier can in principle be used to try to change beliefs. One theoretical framework that appears especially suitable to the health context is Protection Motivation Theory (PMT) [60]. PMT posits that a threat’s severity and vulnerability on the one hand and response efficacy and self-efficacy on the other hand influence whether people take a recommended health action. Applied to people’s beliefs about the usefulness of competencies for quitting smoking, it is thus the severity of and vulnerability to the consequences of not building a competency as well as the effectiveness of and self-efficacy for building the competency that influence whether people want to build the competency. Using PMT to create persuasive activities, our second analysis question is as follows:

*AQ2: How effective are persuasive activities in changing usefulness beliefs?*

In the following, we describe our five pipeline steps for building our RL model for proposing smoking cessation activities as shown in Fig. 1.

## Methods

### Step 1: Establishing competency-building activities

The first step was to understand which activities are currently used by health experts to prepare smokers for quitting smoking. These activities build the competencies for quitting smoking that health experts consider relevant, even if the competencies have not been standardized. Based on discussions with health experts from the network of our project, primarily with a background in Psychology, the activities by Albers et al. [57], the behavior change techniques by Michie et al. [61], and smoking cessation material by organizations such as the National Cancer Institute and the Dutch Trimbos Institute, we obtained 44 preparatory activities (e.g., envisioning quitting smoking as a fighting match, thinking of past successes, or writing a positive diary). Some activities addressed becoming more physically active since this can make it easier to quit smoking [55, 56]. A health psychologist and smoking cessation expert checked the activities to ensure they were suitable and clear. Table S1 in the Appendix [27] lists all preparatory activities.

### Step 2: Obtaining the views of health experts and smokers

**Fig. 2:**
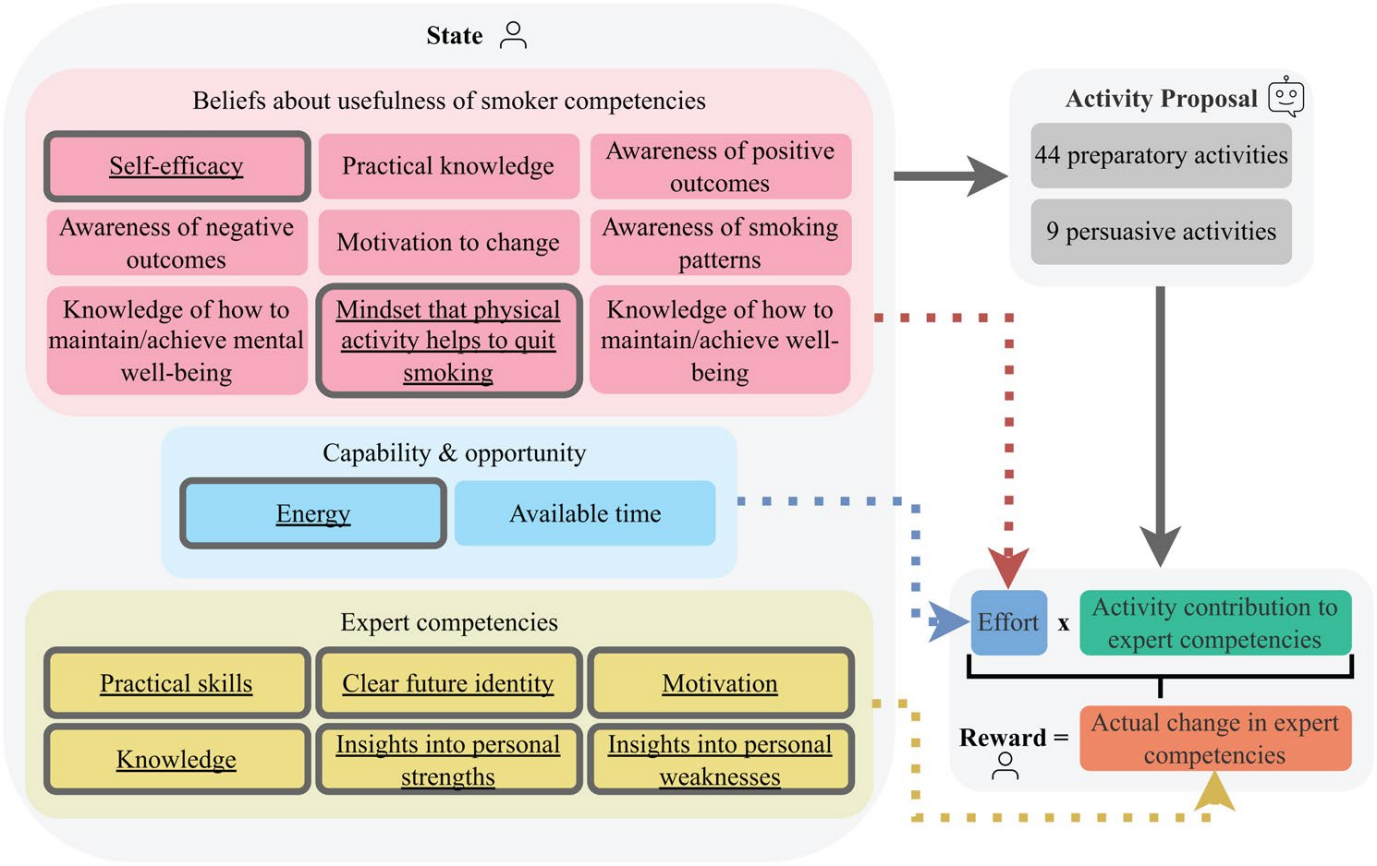
Visualization of the RL model. The state features with thick borders are used in the final trained model

Having established the 44 preparatory activities, the next step was to determine how health experts and smokers view them. The "views" in our case are the two sets of competencies for quitting smoking that experts and smokers think are built by the activities. If we know which competencies the activities build according to *experts*, our model can keep track of the extent to which smokers have already built the different competencies (yellow rectangles in Fig. 2) and choose activities that help build missing competencies. On the other hand, by knowing which competencies *smokers* think are built by the activities, our model can consider which competencies smokers find useful when choosing activities (red rectangles in Fig. 2).

To this end, we conducted two repertory grid studies, one with experts and one with smokers. Based on personal construct theory [62], the goal of the repertory grid technique is to explore personal construct systems, or, in other words, see the world as other people see it [63]. The people whose world one would like to see, in our case experts and smokers, were given three preparatory activities and asked to divide them into two groups based on considering how two activities are alike in some way but different from the third activity. After providing a label for each resulting group, participants rated each of the 44 activities on a seven-point scale from "not at all related to $\langle label\rangle$" to "strongly related to $\langle label\rangle$" for each label.

For each repertory grid study, these ratings served as input for an exploratory factor analysis with an oblique rotation, minimum residuals as extraction method as recommended by Izquierdo et al. [64], and the common cutoff value of 0.4 [65]. For each possible number of factors identified with the scree method and parallel analysis, we examined the resulting factors according to their theoretical and practical plausibility. The final factors describing the views of experts and smokers were chosen based on this examination by two researchers (N.A. and either W.B. or K.P.). All our factors satisfy the recommendation that independent of the sample size, factors are reliable as long as the average of the four largest loadings is greater than 0.60 [66].

The studies were preregistered in the Open Science Framework [67] and approved by the Human Research Ethics Committee of Delft University of Technology (Letter of Approval number: 2338, date: 27 June 2022). All participants gave digital informed consent. Below we provide more information on the two studies and their results.

#### View of health experts

First, we investigated which competencies the preparatory activities build according to experts.

*Approach.* Rather than using a single expert as is sometimes done when developing expert systems [68], we had the opportunity to reach multiple experts from the network of our project to account for possible biases, narrow lines of reasoning, and incomplete domain expertise [68]. Specifically, we conducted a repertory grid study with four smoking cessation experts who described their backgrounds as "psychology" (*N* = 2), "health and medical psychology" (*N* = 1), and "general practitioner" (*N* = 1). Since our project focuses on using physical activity as an aid for quitting smoking, it was possible to reach people with expertise on both behaviors. On a scale from 0 ("No expertise at all") to 10 ("Extremely strong expertise"), the experts reported having strong expertise in coaching for behavior change (*M* = 8.25, *SD* = 0.96), coaching for quitting smoking (*M* = 8.25, *SD* = 0.50), and coaching for becoming more physically active (*M* = 7.50, *SD* = 1.00). Each expert was asked to do the task four times, each time with a new set of preparatory activities, using the question "When it comes to competencies for quitting smoking that smokers build by doing the activities, how are two activities alike in some way but different from the third activity?" To ensure that the experts understood the question, they had to pass an attention check on the question’s meaning after being provided with both a definition and an example.

*Results.* The exploratory factor analysis on the 32 items (i.e., labels and corresponding activity ratings) led to three factors: 1) practical skills – clear future identity, 2) motivation – knowledge, and 3) insights into personal strengths – insights into personal weaknesses. The six factor endpoints gave us six individual competencies for quitting smoking (yellow rectangles in Fig. 2). Table 1 provides examples of the labels and explanations by experts mapped to the first factor. For examples for all three factors refer to Table S3 in the Appendix.

**Table 1:** Factor loadings, labels, and explanations of the three items with the most positive and negative factor loadings for two of the factors found through the repertory grid studies (i.e., one factor from the repertory grid study with health experts and one factor from the study with smokers)

Factor for expert-identified competencies: (+) practical skills – clear future identity (−)	
0.85	practical skills: preparing practically for activities
0.80	strategies: through social learning the individual might find out strategies to successfully change behavior themselves
0.78	problem solving: This helps smokers to think ahead and come up with solutions for barriers
-0.63	Identity: These activities help to strengthen feared and ideal future selves
-0.63	identity: These activities help to envision the ideal and feared futire selves
-0.64	Future-self: Future-selves can act as powerful motivators
Factor for smoker-identified competencies: (+) self-efficacy – practical knowledge (−)	
0.92	Self motivation: Activities that focus on motivation and planning
0.85	Mindset: Activities that will help you with the right mindset needed to quit smoking
0.79	Mindsets: getting into the right mindset
-0.75	Problem solving: Quitting smoking and being more active can some times be hard and some barriers may show up…
-0.84	Knowledge: Consuming educational content, gaining knowledge
-0.86	Pratical: Learning real techniques to quit smoking

The labels and explanations are direct, uncorrected quotes from participants

#### View of smokers

Next, we explored smokers’ views on competencies for quitting smoking built by preparatory activities.

*Approach.* Aiming for 4 participants per combination of values for age range (3 levels), gender (2 levels), weekly exercise amount (3 levels), and smoking frequency (2 levels), we conducted an online crowdsourcing study with $4 \times (3 \times 2 \times 3 \times 2) = 144$ daily smokers who were contemplating or preparing to quit smoking [69]. Each participant received two sets of three preparatory activities. 76 participants were instructed to divide the activities in a set into two groups based on the question "When it comes to competencies for quitting smoking that smokers build by doing the activities, how are two activities alike in some way but different from the third activity?" The other participants were asked to divide the activities based on what people have to do for an activity (e.g., visualize, record) for future research. For each resulting group, participants provided a label as well as an explanation of the label. To increase the validity of the data, participants had to pass a multiple-choice attention check question on the meaning of competencies or doing something for an activity after seeing both an explanation and an example. Based on our observations from two small pilot studies, we suspected that not all participants followed the instructions. Therefore, the first author coded all obtained labels as 1) competency (*N* = 153), 2) a way of doing a preparatory activity (*N* = 254), or 3) unclear (*N* = 169) by looking at both the labels and their explanations. To examine the reliability of the coding, we made use of a second coder (M.T.). The first author trained this second coder by explaining the coding of 12 example labels and giving feedback on six rounds of coding ten labels. Based on the subsequent independent coding of 100 labels by the second coder, we obtained a Cohen’s *κ* of 0.55 and a Brennan-Prediger *κ* of 0.56, indicating moderate agreement [70]. Finally, in the exploratory factor analysis, we included only those activity ratings whose labels the first coder had coded as competency. Table S2 in the Appendix shows the participant characteristics.

*Results.* We obtained five factors from whose endpoints we created nine competencies (red rectangles in Fig. 2)^1^. For example, we created the competencies "self-efficacy" and "practical knowledge" from the first factor (Table 1).

### Step 3: Creating persuasive activities

If the virtual coach knows which smoker-identified competencies a person finds useful, it can propose competency-building activities that they find more useful. However, the virtual coach can also try to change the person’s usefulness beliefs about competencies, especially when building all expert-identified competencies is otherwise not possible because the person finds all related activities not useful. We, therefore, designed nine persuasive activities. Together with the 44 preparatory activities designed in step 1, the virtual coach can thus choose from 53 activities during each interaction. While the *preparatory* activities are meant to directly build competencies, each *persuasive* activity is meant to first persuade smokers of the usefulness of one of the nine smoker-identified competencies so that they will later spend effort on corresponding activities that build competencies. As we worked with two different worldviews (i.e., of smokers and experts) we accepted to some extent that smokers might do the "right" thing for the wrong reasons. Still, we first verified that each smoker-identified competency could be mapped to one or more expert-identified competencies (e.g., "self-efficacy" could be mapped to "motivation" and "insights into personal strengths"). This ensured that the content of the persuasive activities was also grounded in the views of the experts. Each persuasive activity was then built to persuade people of the usefulness of one smoker-identified competency by addressing elements from PMT (e.g., see Table S5 and Table S6 in the Appendix). A health psychologist and smoking cessation expert read through all activities to ensure that they were suitable and clear. The nine persuasive activities can be found in Table S7 in the Appendix.

### Step 4: Designing the model

Next, we designed a model that a virtual coach can use to choose activities. We can define our approach as a Markov Decision Process (MDP) $\langle S, A, R, T, \gamma \rangle$. The action space *A* consisted of 53 activities (i.e., 44 preparatory and 9 persuasive activities), the reward function $R: S \times A \rightarrow [0, 6]$^2^ was determined by the self-reported effort spent on activities and by the activities’ contributions to the expert-identified competencies, $T: S \times A \times S \rightarrow [0, 1]$ was the transition function, and the discount factor *γ* was set to 0.85 to favor rewards obtained earlier over rewards obtained later due to the importance of initial small wins [71]. The finite state space *S* described the state a person was in and was captured by their beliefs about the usefulness of smoker-identified competencies, their capability and opportunity, and their levels for expert-identified competencies. The goal of an agent in an MDP is to learn an optimal policy $\pi^*: S \rightarrow \Pi(A)$ that maximizes the expected cumulative discounted reward $\mathbb{E}\big[\sum_{t=0}^\infty \gamma^tr_t\big]$ for acting in the environment. The optimal Q-value function $Q^*: S \times A \rightarrow \mathbb{R}$ describes the expected cumulative discounted reward for executing action *a* in state *s* and $\pi^*$ in all subsequent states. Fig. 2 visualizes the model, the components of which are described in more detail below.

#### State space

The state space had three components: 1) people’s beliefs about the usefulness of the nine smoker-identified competencies, 2) their capability and opportunity for doing an activity, and 3) their levels for the expert-identified competencies. We included people’s capability and opportunity as they predict behavior according to the COM-B model [52] alongside motivation, which was captured by the usefulness beliefs.

To infer a person’s state, the virtual coach would ask questions during its interaction with them. For the usefulness beliefs, people would answer nine questions after the prompt "Please rate how you think the following 9 factors affect quitting smoking. Answer on a scale from -10 to 10, where −10 indicates that quitting smoking is made a lot harder and 10 indicates that quitting smoking is made a lot easier. 0 indicates ŉeutral’." in each session with the virtual coach. To ensure that the questions are understandable for smokers, we used the terminology smokers used in the repertory grid study together with some specific examples they gave (Table S8 in the Appendix). For example, for the competency "practical knowledge," we used the formulation "practical preparation (e.g., learning how to relieve stress, knowing effects of nicotine, getting organized)."

To measure people’s capability and opportunity to do preparatory activities, the virtual coach would further ask people about their energy and available time on 11-point scales.

Lastly, people’s levels for the six expert-identified competencies (i.e., degrees of having built these competencies) were initialized to 0 and subsequently updated to a value in the set $\{0, 0.33, 0.67, 1\}$ to obtain a reasonably sized state space. The updating process is described in more detail for the transition function.

#### Action space

There were 53 actions: the 44 preparatory activities for quitting smoking and the 9 activities meant to persuade people of the usefulness of the smoker-identified competencies for quitting smoking.

#### Reward

The intuition behind the reward signal is that people should ultimately build the competencies identified by experts, that these competencies are only built if people do their activities thoroughly, and that there is an upper limit to building each competency (i.e., at some point, a competency has been fully built). The idea thus is that the reward captures the *actual* increase in these competencies. Therefore, the reward was, accounting for an upper limit of 1 for each expert-identified competency, the product of two measures: 1) an activity’s contribution to the expert-identified competencies, and 2) the effort people spent on the activity as a measure of their engagement with it. Engagement and competency development have previously been linked in educational contexts (e.g., [72]). Since the first measure is based on the experts’ perspective and the second measure on the smokers’ perspective, this reward signal combines the two perspectives.

For the first measure, we computed the contribution of each preparatory activity to the expert-identified competencies based on the factor loadings from the repertory grid study with experts, scaled to the interval $[0, 1]$ (Table S9 in the Appendix). The contributions of the persuasive activities were set to 0 as these activities do not build any competencies but only aimed at changing usefulness beliefs. For the second measure, people were in each session asked about the overall effort they spent on their last activity on a scale from 0 to 10, adapted from Hutchinson and Tenenbaum [73] as also used by Albers et al. [25]. The effort responses were also scaled to the interval $[0, 1]$, with the population-level mean effort mapped to 0.5 so that values for efforts greater and lower than the mean were each equally spaced. To reduce the amount of required data, we grouped the preparatory activities into five clusters to predict the effort. To this end, we performed k-means clustering using the smokers’ ratings of the preparatory activities’ contribution to smoker-identified competencies from the repertory grid study. This means that preparatory activities seen as contributing similarly to the smoker-identified competencies were grouped together (Table S1 in the Appendix). Note that we opted for this clustering based on *smokers’* perceptions of the activities since we assume in our model that it is smokers’, rather than experts’, perceptions of the activities that influence how much effort smokers spend on the activities.

**Fig. 3:**
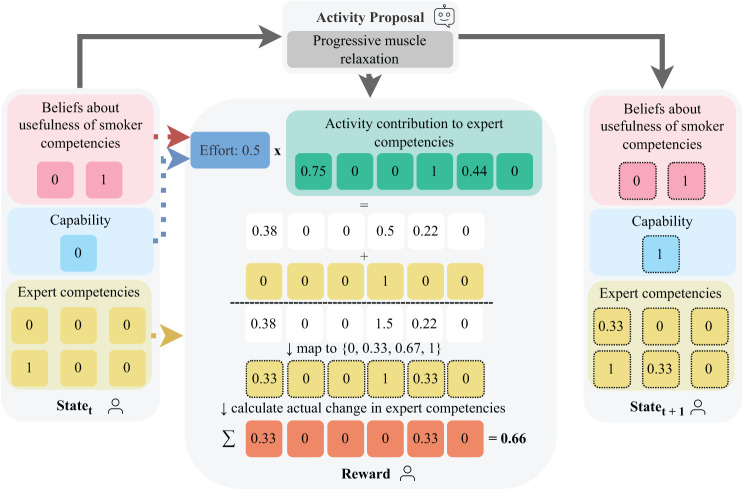
Example of how the reward of 0.66 and the next expert competency levels are computed after a person spent an effort of 0.5 on the activity "progressive muscle relaxation"

Given a maximum value of 1 for each expert-identified competency, the *actual* increase in the expert-identified competencies for person *i* after spending effort $e_{a, i}$ on activity *a* was then calculated as $\sum_{j=0}^{5} min\{pc_{a, j, i}, 1 - comp_{j, i}\}$, where $pc_{a, j, i}$ is the possible contribution $cont_{a, j} \times e_{a, i}$ mapped to the possible levels for the expert competencies $\{0, 0.33, 0.67, 1\}$, $cont_{a, j}$ is the contribution of activity *a* to expert-identified competency *j*, and $comp_{j, i}$ is the current level of competency *j* for person *i* (Fig. 3). Note that since the preparatory activities are only clustered for predicting one component of the reward (i.e., the effort), the reward can be different for each of the 44 preparatory activities.

#### Transition function

The transitions between values for the user-inquired state features (i.e., the usefulness beliefs and people’s capability and opportunity) were learned from data, whereby transitions for one state feature were considered independent of the values of other features. To reduce the amount of data required to reliably predict the transitions, the preparatory activities were grouped into the same clusters as for the effort prediction when predicting the next usefulness beliefs and people’s capability and opportunity. People’s levels for the six expert-identified competencies, on the other hand, were updated up to a maximum value of 1 based on 1) an activity’s contribution to the expert-identified competencies according to the data from the repertory grid study with experts and 2) the effort people spent on the activity, mapped to the interval $[0, 1]$. The raw updated level of person *i* for an expert-identified competency *j* after spending effort $e_{a, i}$ on activity *a* was then computed as $comp_{j, i, t+1} = comp_{j, i, t} + e_{a, i} \times cont_{a, j}$, where $cont_{a, j}$ is the contribution of activity *a* to expert-identified competency *j* (Fig. 3). The resulting raw value was then mapped to the closest value in $\{0, 0.33, 0.67, 1\}$ to get the next value for the state feature. The contributions of all preparatory activities to the expert-identified competencies are provided in the Appendix. The nine persuasive activities do not contribute to the expert-identified competencies. Note that the change in levels for the expert-identified competencies also informed the reward computation.

### Step 5: Training the model

To train our model, we conducted a study in which daily smokers interacted with the virtual coach Mel in five sessions, which were spread over at least nine days to give people at least two days to complete their activities between sessions. To facilitate training the model with the collected data, the study had a micro-randomized design [74] where Mel proposed randomly chosen activities in each of up to five sessions, which means that each participant was randomized up to five times. Ultimately, however, we envision Mel using our trained model to choose activities.

*Virtual coach.* We implemented the text-based virtual coach Mel. Mel introduced itself as wanting to prepare people for quitting smoking and becoming more physically active, with the latter possibly facilitating the former. In each session, Mel determined people’s current state by asking about their beliefs regarding the usefulness of the competencies for quitting smoking identified by smokers as well as their available time and energy. Afterward, Mel proposed a new preparatory or persuasive activity. In the next session, which participants were invited to about two days later, Mel asked about the effort people spent on their activity from the previous session as well as their experience with it. In its conversation structure and style, Mel was closely based on the virtual coach Sam [75], whose scripted dialogs were developed for another smoking cessation study and were overall perceived positively by its users [57, 76]. Like Sam, Mel gave compliments for spending a lot of effort on activities, expressed empathy otherwise, and kept a generally positive and encouraging attitude. The Rasa-based implementation of the virtual coach [77] as well as a demo video [78] are available online. The conversation structure is depicted in the Appendix.

*Study.* We conducted a study in which people interacted with Mel in up to five conversational sessions between 21 July and 27 August 2023. The Human Research Ethics Committee of Delft University of Technology granted ethical approval for the research (Letter of Approval number: 2939) on 31 March 2023. Before data collection, the study was preregistered [79]. Participants were recruited from the crowdsourcing platform Prolific Academic. Eligible were people who were contemplating or preparing to quit smoking [69], smoked tobacco products daily, were fluent in English, were not part of another intervention to quit smoking, had not participated in our repertory grid studies, and gave digital informed consent. To choose a new activity, Mel first randomly chose from the five preparatory activity clusters and nine persuasive activities. If a cluster was chosen, Mel then randomly selected one of the activities mapped to it. For completing each study part, participants were paid based on the minimum payment rules on Prolific (i.e., six GBP per hour). They were also informed that their payment was independent of them completing their activities. 682 people started the first session and 349 people completed session 5 (Fig. S2 in the Appendix). Participant characteristics such as age, gender, and smoking frequency at the start of the study are shown in Table S10 in the Appendix. Two days ($T1$, *N* = 324) and eight weeks ($T2$, *N* = 245) after the last session, participants’ smoking frequency was lower ($T1$ – $T0$: M = −0.67, 95%-HDI = [-0.96, -0.38]; $T2$ – $T0$: M = −0.96, 95%-HDI = [-1.30, -0.63]; 8-point scale) and quitter self-identity^3^ [80] higher ($T1$ – $T0$: M = 0.21, 95%-HDI = [0.15, 0.27]; $T2$ – $T0$: M = 0.10, 95%-HDI = [0.01, 0.19]; 5-point scale) than at the start of the study ($T0$) (see Table S11 in the Appendix).

*Collected data.* We gathered 1710 $\langle s, a, r, s^{\prime}\rangle$-samples from 542 people, where *s* is the state, *a* the action, *r* the reward, and *s*^ʹ^ the next state. Participants spent an average effort of 5.58 (*SD* = 2.86) on their activities, with the mean effort per preparatory activity cluster ranging from 5.30 (*SD* = 2.85) to 6.14 (*SD* = 2.72) and the one per persuasive activity from 5.19 (*SD* = 2.94) to 5.95 (*SD* = 2.66) (Table S12 in the Appendix). In sessions 2–5, participants were asked about their likelihood of having returned to the session in case of an unpaid smoking cessation program on a scale from −5 ("definitely would have quit the program") to 5 ("definitely would have returned to this session"). The mean of these responses was 1.44 (*SD* = 2.74) in session 2 and 1.80 (*SD* = 2.94) in session 5, with responses from the full range of the scale in each session.

*State space reduction.* Even when using only binary state features, using all nine usefulness beliefs and both capability and opportunity features in our model would lead to $2^{11} = 2048$ possible values for those state features that influence dynamics components that we need to estimate from data (i.e., the effort and the transitions between these features). To reduce the size of the state space and hence the amount of required data, we transformed the usefulness beliefs and the capability and opportunity features into binary features based on whether a value was greater than or equal to the sample mean (1) or less than the mean (0). Moreover, we used our data to select three features in a way that was inspired by the G-algorithm [81]. This involved iteratively selecting the feature for which the effort-based Q-values were most different when the feature is 0 compared to when the feature is 1. Besides the reduction in state space size, this selection also has the benefit that users would need to answer fewer questions in practice. The selected features were: 1) belief about the usefulness of "self-efficacy," 2) belief about the usefulness of the competency "mindset that physical activity helps to quit smoking," and 3) energy. The final model had $2^3 = 8$ different values for those state features that influence dynamics components we need to estimate from data as well as $4^6 = 4096$ different values for the expert competency features that influence the dynamics deterministically. The entire state space hence had size $|S| = 8 \times 4096 = 32768$. Fig. S3 in the Appendix shows the mean effort per combination of values for the three selected user-inquired features.

*Model training.* We used the 1710 collected samples to estimate the population-level reward and transition functions for our RL model. Based on these estimated functions, we computed an 0.001-optimal policy^4^ and corresponding $Q^*$ with Gauss-Seidel value iteration from the Python MDP Toolbox. If an optimal activity had already been proposed to a person in the past, an activity with the next highest $Q^*$ was proposed.

## Results

We now investigate our two analysis questions. For each analysis question, we first describe our approach, followed by our findings and the resulting answer. The data and analysis code underlying this paper are available online [27].

### AQ1: Building expert competencies

*Setup.* To examine how each of our model components contributes to building people’s competencies, as seen by experts, we compared the effects of optimal policies of ablated versions of our model that included or excluded specific components. For this, we analyzed results from human data-based simulations, examining each time how 1000 simulated people would progress in their competency development over multiple interactions with a virtual coach that bases its activity advice on a specific policy. To obtain a realistic population, these simulated people were initially distributed across the user-inquired state features following the distribution we observed in the first session of our data collection study. We created ablated versions of our model by removing increasingly more components from the model: first the learned transitions to the next user-inquired feature values (−*uf*^ʹ^), then the transitions to the next expert competency levels (−*ec*^ʹ^), then the current user-inquired feature values (−*uf*), and so forth. The first five optimal policies we compared, we computed based on these five model versions: 1) the full model ($\pi^*$), 2) assuming that all next user-inquired feature values are equally likely ($\pi^{-uf^{\prime}}$), 3) not considering any future states ($\pi^{-ec^{\prime}, uf^{\prime}}$), 4) considering only a person’s current value for the expert competencies ($\pi^{-ec^{\prime}, uf^{\prime}, uf}$), and 5) considering only current user-inquired feature values by randomly picking one of the activities in the preparatory activity cluster with the highest expected effort ($\pi^{-ec^{\prime}, uf^{\prime}, ec}$). A sixth policy was choosing preparatory activities uniformly at random (*π*^*r*^). Two policies are thus derived from a full RL model ($\pi^*$ and $\pi^{-uf^{\prime}}$), three policies from models that are contextual bandits ($\pi^{-ec^{\prime}, uf^{\prime}}$, $\pi^{-ec^{\prime}, uf^{\prime}, uf}$, and $\pi^{-ec^{\prime}, uf^{\prime}, ec}$), and one policy from a simple baseline model. The five learned optimal policies all differ in some states. For example, the optimal activity indices in the eight possible starting states are $31, 16, 31, 31, 31, 4, 16, 5$ for $\pi^*$, $31, 16, 31, 4, 31, 4, 16, 21$ for $\pi^{-uf^{\prime}}$, and $8, 9, 9, 32, 31, 4, 32, 21$ for $\pi^{-ec^{\prime}, uf^{\prime}}$. None of the five learned policies included a persuasive activity.

**Fig. 4:**
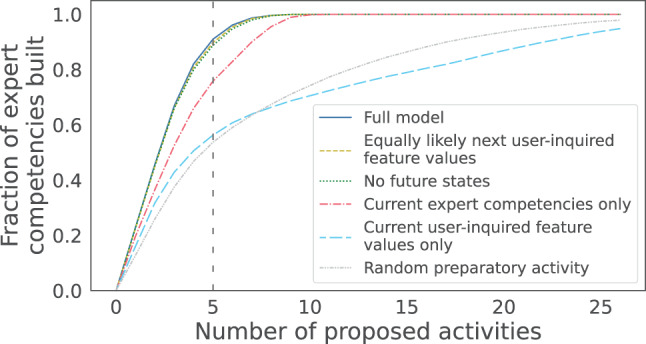
Fraction of expert competencies built after different numbers of proposed activities when using policies based on different models to choose activities. The lines for the first three policies overlap almost completely

*Results.* Using more model components generally allows the expert competencies to be built more quickly (Fig. 4). However, removing the learned transitions to the next user-inquired feature values (*dashed yellow line*) and the transitions to the next expert competencies (*dotted green line*) each leads to at most a small deterioration (1%). Proposing a random preparatory activity or a preparatory activity that people are expected to spend the most effort on based on their current user-inquired feature values performs worst. After five proposed activities, using $\pi^*$ has allowed people to build 91% of the competencies, $\pi^{-uf^{\prime}}$ 90%, $\pi^{-ec^{\prime}, uf^{\prime}}$ 89%, $\pi^{-ec^{\prime}, uf^{\prime}, uf}$ 76%, $\pi^{-ec^{\prime}, uf^{\prime}, ec}$ 56%, and *π*^*r*^ 54%. The largest drops in performance result from removing either the current user-inquired feature values (13%, effect size Cohen’s *h* = 0.34) or the current expert competency levels (32%, Cohen’s *h* = 0.76). These are small to medium effects according to the classification guidelines by Cohen [82]. These results are relatively robust to small variations in the training data, as the repeating of the analysis when training the models based on different subsets of the interaction samples shows (Fig. S5 in the Appendix). For example, all three tested subsets of 95% of the interaction samples per action mean that the use of $\pi^*$ allows smokers to build 90% of the competencies with five proposed activities.

*Answer to AQ1.* With just five proposed activities, an RL model that combines the views of experts and smokers allows smokers to build 91% of the expert-identified competencies our preparation program can teach. 54% could be attributed to assigning any preparatory activity, 22% to considering current levels of expert competencies, 13% to also considering smokers’ current usefulness beliefs and energy, and 1% each to further considering future levels of expert competencies and smokers’ future usefulness beliefs and energy.

### AQ2: Changing usefulness beliefs

*Setup.* Our analysis for *AQ1* showed that considering smokers’ current usefulness beliefs helps to choose activities that build expert competencies more quickly. This suggests that people’s usefulness beliefs impact the effort they spend on preparatory activities (see also Fig. S3 in the Appendix). It would thus be beneficial if we could change people’s usefulness beliefs so that people spend more effort on activities. In *AQ1*, we already saw that optimal policies propose only preparatory and not persuasive activities. So persuasive activities are likely not as effective in changing people’s usefulness beliefs as we envisioned them to be. To investigate whether there is any effect of the persuasive activities on the usefulness beliefs, we performed paired Bayesian *t*-tests using the Bayesian First Aid package [83], comparing the usefulness belief corresponding to a persuasive activity before and after people were assigned the activity.

*Results.* Table 2 shows that all persuasive activities positively impact the corresponding usefulness beliefs with a posterior probability of at least 0.84. For two activities, this probability is even > 0.9995, which can be evaluated as "nearing certainty" that the effect is positive [84]. Effect sizes (Cohen’s *d*) thereby range from 0.01 for "practical knowledge" to 0.45 for "awareness of negative outcomes" and are hence less than small to at most small according to the classification guidelines by Cohen [85].

**Table 2:** Mean impact of the nine persuasive activities on the corresponding usefulness beliefs based on paired Bayesian *t*-tests

Competency	Mean (SD)	95% HDI	Prob > 0
Self-efficacy	0.35 (0.17)	[0.01, 0.69]	0.98
Practical knowledge	0.17 (0.18)	[-0.18, 0.52]	0.84
Awareness of positive outcomes	0.31 (0.15)	[0.00, 0.61]	0.98
Awareness of negative outcomes	1.39 (0.52)	[0.45, 2.42]	>0.9995
Motivation to change	0.58 (0.28)	[0.05, 1.13]	0.99
Knowledge of how to maintain/achieve mental well-being	0.40 (0.18)	[0.04, 0.76]	0.99
Mindset that physical activity helps to quit smoking	0.43 (0.20)	[0.04, 0.81]	0.99
Awareness of smoking patterns	1.16 (0.27)	[0.63, 1.70]	>0.9995
Knowledge of how to maintain/achieve well-being	0.23 (0.21)	[-0.18, 0.63]	0.87

Abbreviations: SD, Standard deviation; HDI, Highest density interval

*Answer to AQ2.* While persuasive activities overall do have a positive effect on the usefulness beliefs, the effects seem to be too small for optimal policies to suggest them instead of actual preparatory activities. This might at least be the case when user-inquired features are binary as in the case of our simulation.

## Discussion

We have presented a five-step pipeline for creating an RL model for building human competencies for quitting smoking that combines the views of health experts and smokers. To train the model, we conducted a crowdsourcing study with 542 daily smokers doing randomly chosen preparatory and persuasive activities for quitting smoking in up to five sessions. It is interesting to point out that even though participants only did *random* activities, their quitter self-identity was somewhat higher and their smoking frequency lower than before the study both two days and eight weeks after the last session. There is some evidence from waitlist conditions in random waitlist-controlled trials that quitter self-identity [86] and smoking frequency [87–89] remain relatively constant if there is no intervention. While this suggests that already doing randomly chosen preparatory activities might increase quitter self-identity and reduce smoking frequency in smokers, caution is required as external factors such as time could have contributed to the observed effect.

Based on the data from the study, we performed simulations to assess the benefit of each RL model component in building expert-identified competencies in smokers. Within just five interactions with the virtual coach, proposing activities based on the full model can allow smokers to build 91% of the expert competencies our preparation program can teach (*AQ1*). Compared to the 54% of expert competencies built by proposing five random preparatory activities, this is an increase by a factor of almost 1.7. All model components contribute to this. People’s current state based on both their levels for the expert competencies and their usefulness beliefs and energy is most important. In fact, we saw improvements of up to 22% when considering current competencies, and an additional 13% when also accounting for current usefulness beliefs and energy. The contributions of the learned transitions to the next user-inquired feature values and the transitions to the next expert competencies, on the other hand, are small. This confirms the value of considering, if not the *future*, at least the *current* views of smokers and experts. In line with the finding by Doroudi et al. [90] that especially RL based on ideas and theories from cognitive psychology and the learning sciences helps to optimize instructional sequencing, this shows the benefit of a psychology-informed model which, analogously to physics-informed algorithms incorporating physical laws to facilitate learning [91], includes psychological information.

Given that considering the transitions to next values for the user-inquired features hardly contributes to building expert competencies, it seems that the effect of *preparatory activities* on usefulness beliefs is small. Our analysis of the effects of *persuasive activities* indicates the same for these activities (*AQ2*). Specifically, while there is a high probability that all persuasive activities positively impact the corresponding usefulness beliefs, the effects are too small for an optimal policy to suggest persuasive instead of preparatory activities. Future research could examine the effects of other (e.g., testimonials [92]) or refined (e.g., via a human-centered design approach as done by Walji and Zhang [93]) persuasive activities. Yet, our findings are in line with the often small effects of individual persuasive attempts on behavior (e.g., [25, 48, 94]). Multiple persuasive attempts might hence be needed to clearly change a usefulness belief. As long as users’ current usefulness beliefs still make them do activities that eventually help them build the expert competencies, however, it might be more effective for the virtual coach to focus on proposing activities that people already regard as useful than trying to change usefulness beliefs. At least in our simulations, people still succeed in building the expert competencies. If this turns out not to be the case in practice, or if an intervention developer cares about changing usefulness beliefs in their own right, a measure of belief changes could be added to the reward signal. Such an adaptation of the reward signal might also be accompanied by a reconsideration of the discount factor, which we had chosen based on theoretical considerations related to making small wins, but whose value turned out not to be decisive (Fig. S7 in the Appendix) due to future states playing a small role.

### Limitations regarding competency-building

While the people in our simulations thus still build the expert competencies, our data is from a study in which participants were paid for completing the sessions in which they were assigned activities. Even though participants were informed that their payment was not contingent on completing the activities, they might have felt at least some obligation to do the activities. In a real-world application without such payments, participants who do not find the assigned activities useful might simply drop out and thus never build the expert competencies. This is supported by the observation that in each session some of our participants said that they would definitely have quit the program if it was unpaid. Future work could incorporate these dropout responses into the reward signal, but additional engagement-enhancing strategies, such as feedback from a human coach [95], might also be needed to keep people engaged in a full, unpaid smoking cessation intervention. Furthermore, our simulation was based on the average effort spent on activities in certain states. However, there might be individuals who sometimes or generally spend very little effort on activities. Given our binned expert competency levels, such individuals might only very slowly or never build any expert competencies in our model (e.g., see Fig. S6 in the Appendix). We chose these competency levels as a trade-off that makes our approach computationally feasible, interpretable, and still capable of tracking relevant competency changes. However, future work could investigate more fine-grained competency tracking. More generally, the way we modeled the degrees to which people have built the expert competencies is, of course, a simplification. For example, while we considered expert competencies to be monotonically non-decreasing, competencies such as motivation could in practice also decrease. Moreover, we cannot be sure that somebody who has spent the highest possible effort on an activity has fully learned what the activity was meant to teach. How to improve our model of competency development is an interesting direction for future work, especially given that typical ways of measuring competencies such as taking performance samples [96] do not seem to be suitable for our context. Potentially, a partially observable approach similar to the one taken by Çelikok et al. [97] could be worthwhile to explore. Notably, while more objective measures of engagement than self-reported effort could also be desirable to better judge how well individuals have built competencies in our current model, it is not clear how these can be obtained for all activities (e.g., when an activity is about printing and putting up a picture).

### Data-related limitatons

Besides the reliance on crowdsourced data, our work has several other data-related limitations. First, due to the high cost of collecting human data like ours, we obtained a relatively limited dataset of 1710 samples. We thus turned our user-inquired features into binary features and used only a subset of usefulness beliefs as state features. It could be that using more values for more usefulness beliefs could capture the small positive effects that persuasive activities have on usefulness beliefs. However, a larger dataset is necessary to reliably capture such effects. Notably, using more usefulness beliefs in the model would require asking users more questions in each session, which might be more effortful [98] and thus affect technology use negatively [18]. Second, we grouped preparatory activities perceived similarly by smokers into clusters to more reliably predict the effort and the transitions to values of user-inquired features. A larger dataset could also allow one to remove this clustering and instead capture the effects of individual preparatory activities. Further capturing individual (e.g., [48, 99, 100]) or trait-based (e.g., [25, 39]) differences between smokers might also be worthwhile. This might especially be the case when the participant pool is expanded by, for instance, also including participants in stages of change for quitting smoking other than the contemplation and preparation stage. Lastly, since we took an offline RL approach, our insights are dependent on our dataset [101]. Although human data-based simulations are a common way to assess RL models [20, 26], now that our RL models have been shown to be promising, future work should compare policies trained based on different model components in a randomized controlled trial with activities assigned to real people to see how well our insights from the simulations generalize. This trial could also test how well our findings generalize to a less educated sample. For instance, our knowledge-building activities might be less effective for such a sample due to lower digital health literacy [102] in general and also higher susceptibility to health misinformation [103]. In addition, generalization from preparing for quitting smoking to a complete smoking cessation intervention could be tested. Such an intervention might include additional behavior change techniques (e.g., from those defined in the taxonomy by Michie et al. [104]), which might build further competencies to keep track of, for example as captured by mechanisms of action [105].

### Modeling assumptions

Regarding our model formulation, one limitation further is that our model did not capture delayed effects of activities beyond the next state that could arise because it takes people more time to thoroughly reflect on the activities and change their usefulness beliefs accordingly. Defining surrogate rewards could be a way to address this (e.g., [46]). Moreover, we used domain knowledge to incorporate structure into our RL model and create a relational decomposition that specifies relations between model components [101]. This reduces the amount of data needed to train the model, but limits what can be learned. For example, while we specified that the effort does not depend on the expert competency levels, it could be that building one competency depends on other competencies (e.g., as in educational systems [106] or games [107]). Similarly, it could be that, contrary to our modeling assumptions, changes in different usefulness beliefs do not occur independently. Future work should examine how well our modeling assumptions hold. Furthermore, while constructivism posits that each individual has their own personal construct system with which they see the world [108], we defined a joint construct system to capture the view of all smokers on preparatory activities for quitting smoking. Intuitively, however, the construct systems of individual smokers might differ as they are shaped by personal and smoking-specific experiences (e.g., previous quit attempts). Examining these differences in the future would be interesting. Lastly, our model did not account for some activities having a logical order (e.g., first tracking one’s behavior before considering what to change).

## Conclusions

To help a virtual coach propose effective activities, we have presented an RL model for building human competencies for quitting smoking that combines the worldviews of health experts and smokers. Simulations based on data from a multi-part study with 542 daily smokers support the use of both worldviews in the model, with small to medium effects for smokers’ current usefulness beliefs and energy as well as their current levels for expert competencies. Moreover, while it is possible to positively affect smokers’ usefulness beliefs using short persuasive activities, the effect of these persuasive activities is too small for them to be considered instead of activities that directly aim to build competencies. These findings suggest that it might be more effective to look for the most competency-building activities among the activities people find useful than to try to persuade people of the usefulness of other activities.

## Data Availability

Our analysis code, data, and appendix are available online [27].

## Footnotes

1 As the endpoints of one factor were negations of each other, we created a single competency for that factor.

2 In practice, no activity fully contributes to all six expert competencies, which means that a reward of 6 does not occur.

3 Since the reliability was sufficiently high for $T1$ (Cronbach’s *α* = 0.67, *N* = 324), $T2$ (Cronbach’s *α* = 0.78, *N* = 324), and $T3$ (Cronbach’s *α* = 0.83, *N* = 245), we used the mean of the three items as an index measure.

4 A 0.001-optimal policy means that once the maximum change in the value function for an iteration falls below 0.001, the value function is considered to have converged to the optimal value function, and the resulting value function is then used to compute the optimal policy.

## Abbreviations

MDP: Markov decision process
PMT: Protection motivation theory
RL: Reinforcement learning
JITAI: Just-in-time adaptive intervention
UTAUT: Unified theory of acceptance and use of technology
GBP: Great British pound
